# Anticancer Effects of Sublingual Type I IFN in Combination with Chemotherapy in Implantable and Spontaneous Tumor Models

**DOI:** 10.3390/cells10040845

**Published:** 2021-04-08

**Authors:** Maria Ciccolella, Sara Andreone, Jacopo Mancini, Paola Sestili, Donatella Negri, Anna Maria Pacca, Maria Teresa D’Urso, Daniele Macchia, Rossella Canese, Ken Pang, Thomas SaiYing Ko, Yves Decadt, Giovanna Schiavoni, Fabrizio Mattei, Filippo Belardelli, Eleonora Aricò, Laura Bracci

**Affiliations:** 1 Department of Oncology and Molecular Medicine, Istituto Superiore di Sanità, 00161 Rome, Italy; mariaciccolella06@gmail.com (M.C.); sara.andreone@guest.iss.it (S.A.); jacopo.mancini27@gmail.com (J.M.); giovanna.schiavoni@iss.it (G.S.); fabrizio.mattei@iss.it (F.M.); 2 National Center for the Control and Evaluation of Medicines, 00161 Rome, Italy; paola.sestili@iss.it; 3 Department of Infectious Diseases, Istituto Superiore di Sanità, 00161 Rome, Italy; donatella.negri@iss.it; 4 Animal Research and Welfare Centre, Istituto Superiore di Sanità, 00161 Rome, Italy; annamaria.pacca@iss.it (A.M.P.); mariateresa.durso@iss.it (M.T.D.); daniele.macchia@iss.it (D.M.); 5 Core Facilities, Istituto Superiore di Sanità, 00161 Rome, Italy; rossella.canese@iss.it; 6 Biolingus AG, CH-6052 Hergiswil NW, Switzerland; ken.pang@biolingus.ch (K.P.); thomas.ko@biolingus.ch (T.S.K.); yves.decadt@biolingus.ch (Y.D.); 7 Murdoch Children’s Research Institute, Parkville 3052, Australia; 8 The Walter and Eliza Hall Institute of Medical Research, Parkville 3052, Australia; 9 Department of Paediatrics, University of Melbourne, Parkville 3010, Australia; 10 Institute of Translational Pharmacology, Consiglio Nazionale delle Ricerche, 00133 Rome, Italy; filippo.belardelli@ift.cnr.it

**Keywords:** type I interferon, cyclophosphamide, cisplatin, sublingual delivery, salivary ductal carcinoma, melanoma, lymphoma, multicolor flow cytometry, NeuT transgenic mice, magnetic resonance imaging, immune infiltrates, Her-2, immune response

## Abstract

Salivary gland tumors are a heterogeneous group of neoplasms representing less than 10% of all head and neck tumors. Among salivary gland tumors, salivary duct carcinoma (SDC) is a rare, but highly aggressive malignant tumor resembling ductal breast carcinoma. Sublingual treatments are promising for SDC due to the induction of both local and systemic biological effects and to reduced systemic toxicity compared to other administration routes. In the present study, we first established that the sublingual administration of type I IFN (IFN-I) is safe and feasible, and exerts antitumor effects both as monotherapy and in combination with chemotherapy in transplantable tumor models, i.e., B16-OVA melanoma and EG.7-OVA lymphoma. Subsequently, we proved that sublingual IFN-I in combination with cyclophosphamide (CTX) induces a long-lasting reduction of tumor mass in NeuT transgenic mice that spontaneously develop SDC. Most importantly, tumor shrinkage in NeuT transgenic micewas accompanied by the emergence of tumor-specific cellular immune responses both in the blood and in the tumor tissue. Altogether, these results provide evidence that sublingual IFN holds promise in combination with chemotherapy for the treatment of cancer.

## 1. Introduction

Salivary gland tumors are a heterogeneous group of neoplasms with complex clinical and pathologic characteristics. They represent less than 10% of all head and neck tumors with more than 50,000 new cases reported in 2020 worldwide [1]. Among salivary gland tumors, salivary duct carcinoma (SDC) is an uncommon but aggressive malignant tumor with a high mortality rate, characterized by a high histological resemblance to invasive breast ductal carcinoma [2,3]. Overexpression and/or amplification of proto-oncogene Her-2/neu has been reported in up to 90% of cases, although there is considerable variation between different antibody clones and scoring systems [4,5,6,7]. The role of Her-2/neu overexpression in prognosis and treatment decisions is still controversial [8,9,10]. Regardless of its histological subtype, gold standard treatment of early stage salivary gland tumors consists of surgical resection with or without adjuvant radiation therapy depending on resection margins and nodal involvement [11,12]. In the event of recurrence or metastasis, there is no consensus on the standard of care of salivary gland tumors, including SDC. Cytotoxic chemotherapy, including combinations of cisplatin (CDDP), doxorubicin (DOXO), and cyclophosphamide (CTX), has been poorly effective so far [10,13,14,15]. Instead, targeted treatments are gaining interest based on molecular characterization and subtypes classification (e.g., Her-2+, androgen receptor+) [10,16]. Expression of the programmed death ligand-1 (PDL-1) has been also reported in 30–60% of SDC [17]. Therefore, treatment with immune-checkpoint inhibitors is also being evaluated [18,19].

The search for an effective treatment for SDC has been hampered by the limited availability of mouse models mimicking the human disease [20]. Interestingly, overexpression of the activated Her-2/neu oncogene in mice results in the spontaneous development of mammary duct carcinomas in females [21] and of SDC in males [22]. In a previous report, intratumoral vaccination with a vaccinia virus encoding for ErbB2/Neu (rV-neuT) hampered the growth of transplanted Her-2-expressing SDC in mice [23]. More recently, the combination of Curcumin, a polyphenol with antineoplastic and immunomodulatory properties, with a poxviral vaccine enhanced the antitumoral effect and immune response to Her-2 induced by the rV-neuT vaccine [24], thus highlighting the therapeutic potential of combined immune-based treatments in this malignancy.

Among drugs already in use for salivary cancers, CTX represents a promising candidate for combined therapies, since it combines direct cytotoxic effects and multifaceted immunomodulatory properties [25]. In particular, CTX stimulates a more effective antitumor immune response, by affecting dendritic cell (DC) homeostasis, supporting cytotoxic T lymphocytes activity, modulating Treg/Teffector ratio and triggering the release of immunogenic signals by dying cancer cells [25]. In a previous study from our group, the combination of non-myeloablative doses of CTX with the adoptive transfer of tumor-immune cells and immunoglobulins induced the complete regression of large established breast ductal carcinomas spontaneously arising in NeuT transgenic mice [26]. Of interest, CTX has been shown to synergize with a number of immunotherapies, including type I Interferons (IFN-I) or IFN-I-inducers both in preclinical models and in cancer patients [27,28,29,30,31,32]. In particular, previous data from our group showed that the antitumor effects of CTX were efficiently amplified by IFN-I, the former providing a source of antigen and a “resetting” of the DC compartment and the latter supplying optimal costimulation for T-cell cross-priming, ultimately resulting in the induction of a strong antitumor response and tumor rejection [29].

IFN-I exerts a number of biological functions in cancer disease, including regulation of innate and adaptive immunity and antiangiogenic and proapoptotic effects, making it an obvious anti-cancer treatment. Indeed, IFN-I has been used with some success for the treatment of both hematological malignancies and solid tumors [33,34,35], although the induction of severe side effects, frequently caused by a high dose IFN-I treatments, profoundly limited its clinical use. In the last twenty years, the oromucosal administration of IFN-I has proven to be an effective alternative to standard parenteral administration, allowing the onset of both systemic and mucosal immunity in the absence of hepatic metabolism and proteolytic degradation associated with other administration routes [36,37,38]. Since the sublingual mucosa is highly infiltrated by tolerogenic phagocytes [39,40] and given the immunoactivating effects of IFN-I on DC [29,41,42], the sublingual administration of this cytokine may provide the licensing signal required for T lymphocyte priming against tumor antigens.

In the present study, we investigated the anticancer effect of sublingual IFN-I as a single treatment and in combination with chemotherapy in implantable as well as spontaneous tumor models. The evidences collected from both tumor models confirmed that sublingual IFN-I is safe and feasible, and exerts immune-mediated effects both as monotherapy and in combination with chemotherapy.

## 2. Materials and Methods

### 2.1. Cell Lines and Reagents

EG.7-OVA cells (ATCC^®^ CRL-2113™) and B16-OVA (kindly provided by Drs Andrea Cara and Donatella Negri, Istituto Superiore di Sanità) were routinely checked for chicken ovalbumin (OVA) expression by flow cytometry and RT-PCR. All cells were cultured in RPMI 1640 supplemented with 10% heat-inactivated fetal bovine serum (FBS, Lonza), 2mM L-Glutamine (Lonza), 0.1 U/mL penicillin, 0.1 mg/mL streptomycin (Lonza). At every medium change, Geneticin (G-418 sulphate, 0.4 mg/mL, Gibco) and Geneticin plus Hygromycin-B (0.06 mg/mL, Invitrogen) were added to EG.7-OVA and B16-OVA cells, respectively. The cell lines were routinely tested for the absence of mycoplasma and passaged no more than four times from thawing. Cyclophosphamide (CTX) and cis-diamminedichloroplatinum (II) (CDDP) (Sigma) were dissolved in saline and were filtered sterile before use. Type I Interferons (IFN-I) were produced and partially purified according to a previously published protocol [41]. Since IFN-I preparation originates from the culture supernatant of a L929 murine cell line infected with Newcastle disease virus, supernatants from untreated L929 cells, which contain all the factors spontaneously produced by the cells under steady-state conditions, were employed as a specificity control (mock).

### 2.2. Mice

Seven-to-eight-week-old C57BL/6 (H-2b) female mice (Charles River Italia) were used for experiments with transplantable tumors. 129sv male mice transgenic for the activated rat NeuT oncogene, which spontaneously develop salivary gland tumors (NeuT mice) and NeuT mice carrying a non-functional mutation in the IFN-I receptor (NeuT-IFNAR mice), were generated as previously described [42]. All mice were housed in the animal facility at Istituto Superiore di Sanità in accordance with the European Community guidelines https://eur-lex.europa.eu/eli/dir/2010/63/oj (accessed on 30 March 2021) (Aut n. 107/2016 and further extensions).

### 2.3. Genetic Screening of the NeuT Mouse Colony and Monitoring of Tumors Onset

At each generation, the presence of the transgene was checked by a polymerase chain reaction (PCR) on mice tail DNA using PCR Master Mix (DreamTaq Green) and primers hybridizing to vector (5-ATCGGT-GATGTCGGCGATAT-3) and to MMTV sequences (5-GTAACA-CAGGCAGATGTAGG-3). The reaction was carried out using the Eppendorf Master cycler epgradient S system. Amplified DNA was separated by electrophoretic run on 2.4% agarose gel in Tris-acetate buffer (0.04 M) containing EDTA (1 M) and the fluorescent intercalating Gel Red (Biotium). Transgene-carrying individually tagged males were used in our study. The salivary glands of all transgenic males were inspected once a week to monitor the appearance of the tumor as previously reported [22]. Briefly, tumors were measured twice a week with calipers recording two 2 perpendicular diameters and calculating the mean value. Progressively growing masses bigger that 3 mm mean diameter were regarded as tumors. Mice bearing tumor masses exceeding 20 mm mean diameter or necrotic lesions or mice showing signs of distress were euthanized.

### 2.4. Treatment Protocol

When the tumor size reached 2 mm diameter (B16-OVA) and 10 mm diameter (EG.7-OVA and SDC), mice were injected i.p. with either 5 mg/kg of CDDP or 100 mg/Kg of CTX followed, one day apart, by four daily administrations of IFN-I (7.6 × 10^3^ U) or the same volume of mock as control according to a previously published therapeutic protocol [29]. Mice receiving IFN-I sublingually (Sl-IFN) were anesthetized with 50 µL of Ketavet (43.75 mg/Kg) and Rompum (6.25 mg/Kg) dissolved in sterile saline. The cytokine preparation was admixed 1:1 with a lipophilic sublingual delivery vehicle (Biolingus AG) with the aim of facilitating absorption through the sublingual mucosa. Subsequently, 7 µL of the preparation was placed under the tongue of the animal with a Gilson micropipette. The tongue was massaged with the lateral part of the tip for 30 s before placing the animal in a prone position with the head resting on a support until awakening to avoid swallowing (SOP provided by Biolingus AG). In some experiments, one group of mice received four daily peritumoral injections of IFN-I in combination with CTX or CDDP. Tumor development was measured twice a week with an electronic microcaliper.

### 2.5. Primary Cells

Leukocytes from blood and spleen were collected as described in [43]. Briefly, blood was drawn from the retroorbital plexus and placed in EDTA-coated 1 mL tubes. After and before centrifugation, plasma was collected and stored at −80 °C. The remaining blood cells were diluted in Ammonium-Chloride-Potassium (ACK) lysis buffer (50 mM NH_4_Cl + 10 mM KHCO_3_ + 0.1 mM Na_2_EDTA, pH 7.2–7.4) for erythrocyte lysis before viable count. Spleens, submandibular (smLN) and inguinal lymph nodes (ingLN) were surgically removed from euthanized mice, placed on a cell strainer (70 μm pore size), laid on a sterile Petri dish containing ACK lysing buffer, and gently pressed with a sterile syringe plunger to dissociate the tissue. Splenocyte suspension was incubated for 3 min in ACK lysing buffer to lyse erythrocytes. Complete RPMI was added to block lysis before counting in trypan blue 0.4% solution. Surgically removed tumors were cut into small pieces with sterile scissors before incubation with 1 mg/mL Collagenase Type III (Worthington Biochemical Corporation) and 325 KU/mL DNAse (Sigma) for 30 min at 37 °C. The digested material was filtered on a 70 μm cell strainer and centrifuged before counting in trypan blue 0.4% solution.

### 2.6. IFN-γ ELISpot

Blood leukocytes (10^5^ cells/well) were seeded in triplicate in pre-coated PVDF-96 well plates and incubated with OVA_257–264_ peptide (SIINFEKL, 10 µg/mL, Invitrogen) or Concanavalin-A (Con-A, 5 μg/mL, Sigma) as previously described [43]. Unstimulated wells served as negative control. Spots were counted by using an ELISpot reader (Aelvis).

### 2.7. Flow Cytometry

EG.7-OVA cells were routinely checked for antigen surface expression by flow cytometry after staining with biotinylated anti-Mouse OVA_257–264_ (SIINFEKL) peptide bound to H-2Kb (25-D1.16, ThermoFisher Scientific, Waltham, MA, USA) followed by incubation with streptavidin PE (ThermoFisher Scientific).

Cell suspensions from spleens (2 × 10^5^) or tumors (10^6^) were seeded in 96 well U-bottomed plates and washed twice in staining buffer (PBS + 1% FBS + EDTA 2 mM) before incubation with a viability dye (LIVE/DEAD™ Fixable Near-IR, ThermoFisher Scientific). Samples were washed again in staining buffer and incubated with full FBS to saturate non-specific Ab binding sites. Samples were then stained with the following fluorescent Abs appropriately diluted in staining buffer in appropriate combinations: Anti-c-ErbB2/c-Neu (Ab-4) Ab (7.16.4, Calbiochem); CD45 (30-F11, BD Pharmingen); CD19 (MB19-1), CD3 (17A2), CD8 (53-6.7), CD4 (GK1.5), NK.1.1 (PK136), CD11b (M1/70), CD11c (HL3), F4/80 (BM8), Ly6C (HK1.4), Ly6G (1A8), PD-1 (29F.1A12), Siglec-F (E50-2440) (all from Biolegend); MHC-II (M5/114), CD103 (2E7) (Miltenyi Biotec). Biotinylated Abs were detected by streptavidin BV421 (ThermoFisher Scientific). Cells were resuspended in paraformaldehyde (PFA) 1% and analyzed on a 4-laser flow cytometer (Gallios, Beckman Coulter). Data analysis was performed by using Kaluza™ software (Beckman Coulter). Cells were gated based on forward/side scatter (FSC/SSC) characteristics and their ability to exclude NiR viability dye.

### 2.8. Intracellular Staining

Blood leukocytes (0.5 × 10^6^–1 × 10^6^) were cultured in 96-well U-bottomed plates in complete RPMI in the presence of Brefeldin (1 μL/mL) and Monensin (0.7 μL/mL) and stimulated with MHC-I-restricted Her-2_435–443_ peptide (ILHDGAYSL, kindly provided by Dr Maurizio Federico, Istituto Superiore di Sanità) or with 2 × 10**^−^**^3^ mg/mL Ionomycin (Sigma) plus 2 × 10**^−^**^4^ mg/mL phorbol-12-myristate-13-acetate (PMA, Sigma). After 5 h of incubation at 37 °C and 5% CO_2_, the cells were washed, incubated with the viability dye and surface-stained with fluorescent anti-CD3, anti-CD25, anti-CD8 and anti-PD1. Samples were then fixed and permeabilized (Cytofix/Cytoperm BD) before incubation with anti-IFN-γ (XMG1.2, Invitrogen) and anti-TNF-α biotinylated (Southern Biotech) Abs or matched isotype controls, and streptavidin-BV421 (Biolegend).

For Treg analysis, after surface staining with anti-CD45, anti-CD3, anti-CD4, anti-CD25, cells were fixed and permeabilized before incubation with anti-Foxp3 (FJK-16s, eBioscience) Ab or isotype control (IgG2a) (ThermoFisher Scientific).

All samples were analyzed on a 4-laser flow cytometer (Gallios™, Beckman Coulter). The data were analyzed using Kaluza™ software (Beckman Coulter).

### 2.9. mRNA Extraction and Real Time PCR

Total RNA was extracted from smLN, ingLN and blood of C57Bl/6 mice implanted with B16-OVA and treated with Sl-IFN, Sl-mock and IFNpti for 18 h by using TRIsure reagent (Bioline). mRNA was reverse transcribed by using Tetro cDNA Synthesis Kit (Bioline). Quantitative reverse transcription-PCR (qPCR) with forward and reverse primers for Mx1 and HPRT [44] (Eurofins Genomics) was performed by SYBR Green technology (Sensimix Plus SYBR Kit) (Bioline) by means of an ABI 7500 Real-time PCR system (Applied Biosystems, Thermo Fisher Scientific) and the following reaction conditions: 15 s at 95 °C, 30 s at 60 °C, and 45 s at 72 °C (46 cycles). Triplicates were performed for each experimental point. Data were normalized to HPRT (2-ΔCt method).

### 2.10. Magnetic Resonance Imaging

The experiments were performed using a VARIAN Agilent Inova system for magnetic resonance imaging (MRI) and spectroscopy operating at 4.7 T (Agilent Palo Alto, CA, USA) with a transmitter volume RF coil actively decoupled from the receiver surface coil (RAPID Biomedical). T1-weighted (T1W: repetition time (TR) = 2500 ms, echo time (TE) = 60 ms, thickness = 0.8 mm, FOV 20 × 20 mm^2^, matrix 256 × 128, 21 slices, 4 averages) and T2-weighted MRI (T2W: TR/TE = 2500/60 ms) were acquired on tumors when the dimensions reached or exceeded 500 mm^3^. Diffusion-weighted MRI were acquired in order to allow the measurement of the diffusion and perfusion component of water molecules within the tissue (DWI: TR/TE = 2000/50 ms, thickness = 1.2 mm, FOV 20 × 20 mm^2^, matrix 64 × 64, 12 slices, 2 averages and *b*-values = 0, 31, 69, 99, 200, 314, 707, 1105 s/mm^2^). Water diffusion within tissues was estimated by means of the apparent diffusion coefficient (ADC) parameter that is calculated by using the mono-exponential decay of MRI signals for *b*-values over 150 s/mm^2^. An estimate of the perfusion component was derived from a mono-exponential decay of signals for *b*-values up to 100 s/mm^2^. In addition to the estimation of the average ADC, we performed histogram analysis of the ADC values of every single voxel within the tumor. In particular, we determined the ADCmean, ADCmedian, kurtosis and skewness. Kurtosis measures how sharp is the peak relative to a standard bell curve that indicate how homogeneous the tumor was. Skewness indicates the departure of the histogram profile from horizontal symmetry, which suggests the presence of areas of higher ADC (indicative of necrosis) or lower ADC (proliferating areas) within the tumor. Histogram analyses with their related parameters have been also performed for fast diffusing spins, i.e., for lesser *b* values less up to 100 s/mm^2^ [45]. During the MRI analysis, the animals were anesthetized with isoflurane in a variable percentage between 1.5 and 2.5% in O_2_, at the flow of 1 L/min and positioned on a slide and thermostated at 37.0 °C.

### 2.11. Histology

Major salivary glands from mice at 33 weeks of age (3 mice/group) were excised, fixed in 10% neutral buffered formalin and embedded into paraffin or fixed in 4% PFA and frozen in a cryo-embedding medium (OCT, BioOptica). Five-μm thick slides were deparaffinized, hydrated through graded alcohols and stained with Hematoxylin & Eosin (H&E). Digital images of representative areas were taken by light microscope (Leica).

### 2.12. Statistical Analysis

Unless otherwise specified, results are represented as mean ± SD. A non-parametric Mann–Whitney–Wilcoxon U test was used for group comparisons using Openstat software. The values were considered significant when the probability was below 5% of the confidence level (*p* value < 0.05). Log-rank Mantel–Cox test was used for the analysis of survival curves. For gene expression analysis, one-way ANOVA analysis of variance was performed to compare means among multiple groups, followed by post hoc testing (Tukey).

## 3. Results

### 3.1. In Vivo Anticancer Effect of Sublingual IFN-I

**Figure 1:**
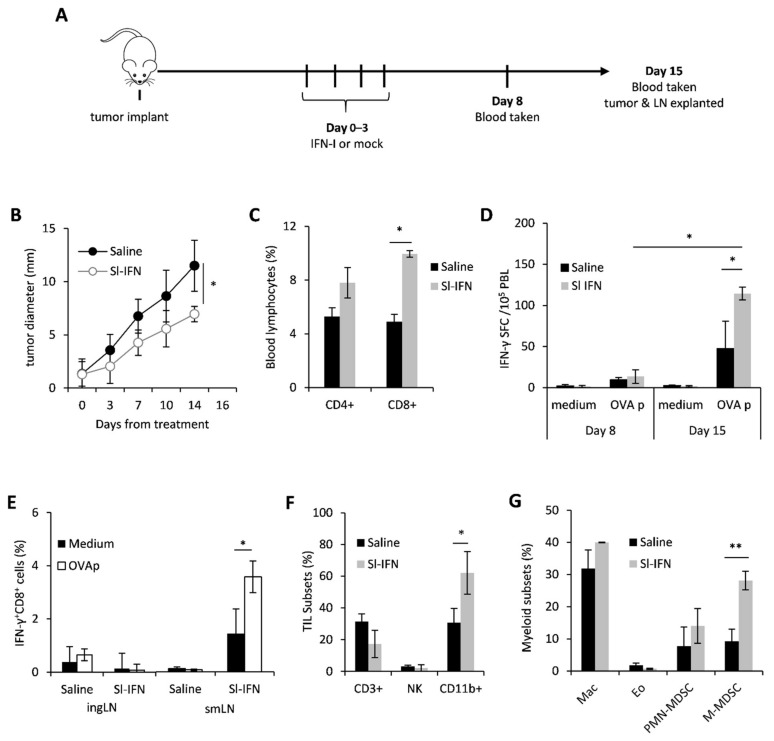
Anticancer effect of sublingual IFN in B16 melanoma. (**A**) Schematic representation of the experimental design. (**B**) Tumor size after sublingual treatment with IFN-I or with Saline as control (*n* = 5). (**C**) Percentage of CD3^+^CD4^+^ and CD3^+^CD8^+^ T lymphocytes in the blood of tumor-bearing mice treated as indicated, 14 days after Sl-IFN treatment initiation (*n* = 5). (**D**) IFN-γ ELISpot in peripheral blood leucocytes (PBL) of C57Bl/6 female mice implanted with B16-OVA treated with Sl-IFN or Saline as control (*n* = 5). Tests have been performed on day 8 and 15 from treatment. (**E**) Percentage of IFNγ^+^CD8^+^ cells in the ingLN and smLN of C57Bl/6 female mice implanted with B16-OVA treated with Sl-IFN or Saline as control (*n* = 5). Assay was performed 15 days after treatment initiation after 5 h stimulation of LN suspensions with OVA peptide (OVAp) or PMA and Ionomycin (PMA/I). (**F**) Percentage of CD3^+^, NK1.1^+^ and CD11b^+^ cells in tumor masses from C57Bl/6 female mice implanted with B16-OVA 15 days after treatment with Sl-IFN or Saline (*n* = 4). (**G**) Percentage of myeloid subsets in the tumor mass of mice treated with Sl-IFN or Saline 15 days after treatment (*n* = 4). * *p* < 0.05, ** *p* < 0.01.

In order to evaluate the anticancer and immunomodulatory activity of sublingual IFN-I in vivo, C57Bl/6 female mice were implanted in the right flank with OVA-expressing melanoma cells (B16-OVA). When tumors became palpable (approx 2 mm), mice were anesthetized and received four daily sublingual administrations of a partially purified IFN-I preparation, thereafter referred to as Sl-IFN, or Saline as placebo (Figure 1A). No signs of toxicity were observed throughout treatment or after treatment completion. On day 8 and day 15 from treatment, blood samples were drawn for the evaluation of antigen-specific immunity by ELISpot. On day 15, mice were euthanized and submandibular LN (smLN), localized above the salivary glands [46], inguinal LN (ingLN), spleen and tumor were excised for the evaluation of immune cell subset composition and functionality by multicolor flow cytometry. Four consecutive administrations of Sl-IFN delayed tumor progression and almost halved tumor size within two weeks from treatment initiation, as compared to controls (Figure 1B). This therapeutic effect was paralleled by a significant increase in the frequency of CD8^+^ T cells in the blood of mice treated with Sl-IFN as compared to Saline-treated animals (Figure 1C). More importantly, CD8^+^ T lymphocytes from mice treated with Sl-IFN produced IFNγ following stimulation with OVA peptide (OVAp) (Figure 1D), although the increase was only detected at day 15. Interestingly, this systemic effect was accompanied by an enrichment of tumor-specific IFNγ^+^CD8^+^T lymphocytes cells in smLN, the lymphoid structure draining the salivary glands, but not in distal ingLN (Figure 1E). Sublingual delivery of IFN-I also induced the accrual of leucocytes into the tumor mass and, in particular, of CD11b^+^ myeloid cells (Figure 1F). Multicolor flow-cytometry analysis of the latter subset revealed the selective increase in monocytic-myeloid-derived suppressor (M-MDSC)-like cells identified as CD11b^+^Ly6G^+^Ly6C^low^ (Figure 1G) in the tumor bed of Sl-IFN treated vs. saline-treated mice. Significant modulations of other myeloid subsets were not observed, including tumor-infiltrating eosinophils (CD11b^+^MHC-II^−^Ly6G^−^Siglec-F^+^) (Figure 1G), whose role in melanoma growth inhibition has been recently reported [47,48,49].

**Figure 2:**
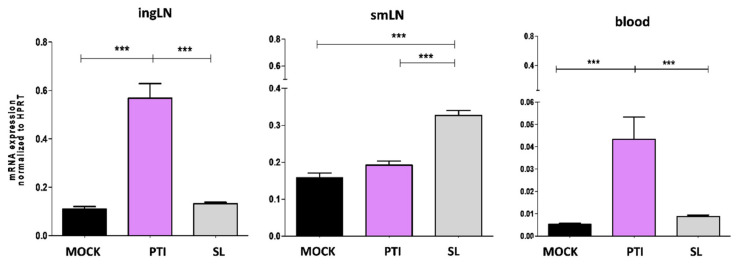
Expression of Mx1 gene by real-time PCR in ingLN, smLN and PBL following sublingual (SL) or peritumoral (PTI) administration of IFN-I or sublingual mock preparation (MOCK) in C57Bl/6 mice implanted with B16-OVA tumor. LNs were explanted 18 h after treatment. Data were normalized to HPRT (*n* = 5). *** *p* < 0.001.

To identify the target organs that respond to sublingual administration of IFN-I, the expression of Mx1, an early hallmark of IFN-I signaling activation, was evaluated in the blood, smLN and ingLN of B16-OVA tumor-bearing mice 18 h after treatment. Sublingual administration of mock (Sl-mock) and the peritumoral administration of the same amount of IFN-I were considered as controls. Interestingly, Mx1 expression significantly increased in the smLN following Sl-IFN administration and in the ingLN following peritumoral IFN-I (IFNpti) (Figure 2) in correlation with the respective administration routes. No change in Mx1 expression was observed in the non-draining LN in any of the treatment groups (Figure 2). Of note, Mx1 upregulation was observed only in the PBL from mice treated with IFNpti, but not with Sl-IFN (Figure 2).

Taken together, these results indicate that Sl-IFN induces both local (i.e., the LN) and systemic (blood, tumor) antitumor responses and that the primary target organs are the smLN.

### 3.2. Anticancer Effect of Sl-IFN in Combination with Chemotherapy in Transplantable Tumors

**Figure 3:**
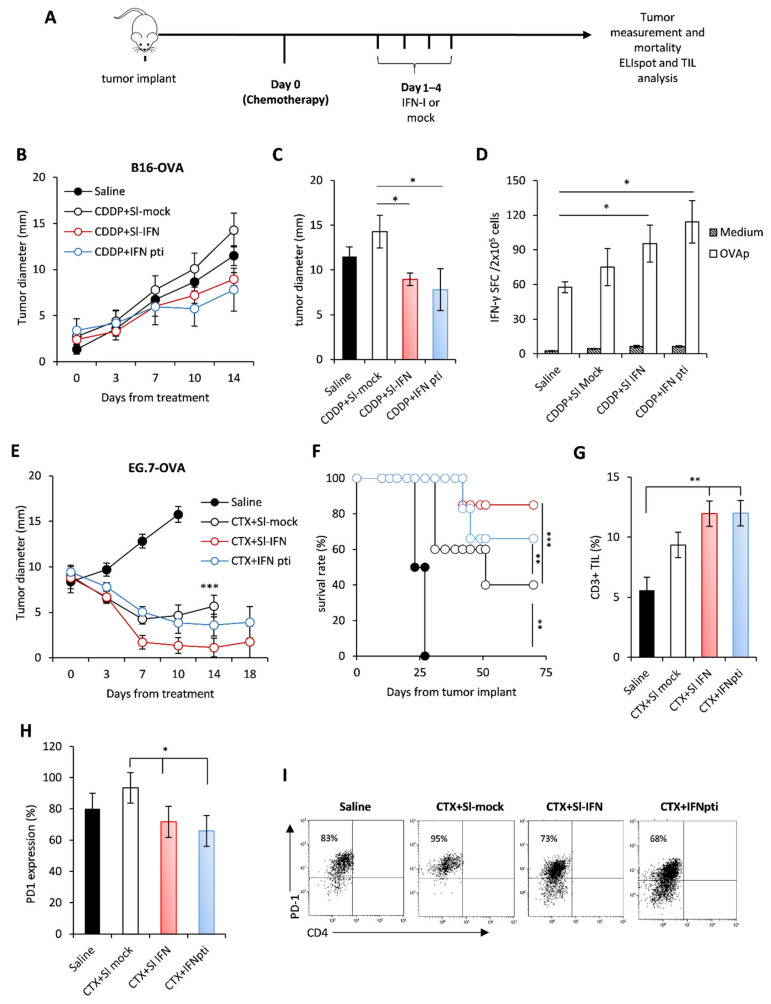
Anticancer effect of sublingual IFN in combination with chemotherapy in transplantable tumors. (**A**) Schematic representation of the experimental design. (**B**) C57Bl/6 female mice were implanted s.c. with B16-OVA on the right flank. On day 10 of tumor growth mice received a single i.p. injection of CDDP followed, 1 day apart, by 4 consecutive administrations of Sl-IFN or IFN pti. One experiment out of two with similar results is shown. * *p* < 0.05 for CDDP + IFNpti and CDDP + Sl-IFN vs. CDDP + Sl-mock. (**C**) Mean tumor size at sacrifice. * *p* < 0.05. (**D**) IFN-γ ELISpot in splenocytes of C57Bl/6 female mice implanted with B16-OVA treated with Sl-IFN or Saline as control (*n* = 5). The test has been performed on day 15 from treatment initiation. * *p* < 0.05. (**E**) C57Bl/6 female mice were implanted s.c. with EG7-OVA on the right flank. On day 13 of tumor growth, mice received a single i.p. injection of CTX followed, 1 day apart, by 4 consecutive administrations of type I IFN either sublingually (Sl-IFN) or peritumorally (IFN pti). One experiment out of two with similar results is shown. *** *p* < 0.001 for CTX + Sl-IFN and CTX + IFNpti vs. Saline. (**F**) Percentage of survival rate. ** *p* < 0.01, *** *p* < 0.005. (**G**) Percentage of CD3^+^ lymphocytes and of (**H**) PD1-expressing CD8^+^ T cells in the tumor tissue 10 days after treatment initiation. * *p* < 0.05, ** *p* < 0.01. (**I**) Representative dot plots of PD-1 expression in CD8^+^ CD4^−^T cells after gating on FSC^lo^CD3^+^ CD45^+^ cells.

Since previous data from our group demonstrated a synergistic anticancer effect of parenteral IFN-I and some anticancer drugs, such as cyclophosphamide (CTX), cis-diamminedichloroplatinum (II) (CDDP) and epigenetic compounds [29,46,50,51], we aimed to evaluate whether Sl-IFN is also effective when combined with these drugs in tumor-bearing mice. Thus, C57Bl/6 female mice were implanted subcutaneously (s.c.) in the right flank with either B16-OVA melanoma cells or with EG.7-OVA lymphoma cells. When tumor masses reached the mean diameter of 2 ± 1 mm and 9 ± 2 mm respectively, mice were injected i.p. with a single dose of CDDP or CTX followed, one day apart, by four daily administrations of Sl-IFN or the same volume of a mock preparation (Sl-mock) as a control. Another group of mice received four peritumoral injections of IFN-I (IFNpti) one day after chemotherapy (Figure 3A), as previously described [29]. As expected, treatment with CDDP had negligible effects on the growth of melanoma tumors (Figure 3B,C) [52,53], while CTX induced a transient reduction of tumor size in EG.7-OVA-implanted mice, leading to cure in 50% of animals (Figure 3E,F). Interestingly, the addition of Sl-IFN to chemotherapy further reduced tumor size and improved mice survival similarly to what was observed in the groups treated with chemotherapy and IFNpti in both tumor models (Figure 3B,E,F). Furthermore, Sl-IFN and IFNpti elicited a slight yet comparable increase in antigen-specific immune responses in the spleen of CDDP-treated B16-bearing mice (Figure 3D) and the accrual of comparable levels of CD3^+^ TIL in the tumor tissue of CTX-treated mice implanted with EG.7-OVA (Figure 3G). Most importantly, the addition of IFN-I decreased PD1 expression on tumor-infiltrating CD8^+^ T (Figure 3H,I), thus suggesting that these cells are less susceptible to PD1-PDL-axis-related immune suppression. Altogether, these data support the concept that Sl-IFN and IFNpti are equally efficient at eliciting antitumor responses in combination with chemotherapy in melanoma and lymphoma models.

### 3.3. Characterization of the Salivary Gland Tumor Model

Since previous data suggest that Sl-IFN preferentially stimulates proximal LN (i.e., smLN), we judged it to be interesting to evaluate the anticancer effect of Sl-IFN, either as monotherapy or in combination with CTX, in male 129sv mice heterozygous for the rat NeuT oncogene (NeuT mice), which spontaneously develop SDC, and to use their IFNAR1 knock-out counterparts (NeuT-IFNAR mice) as specificity controls. As a first step, we assessed whether the lack of a functional IFN-I system would affect spontaneous SDC development and morphology.

**Figure 4:**
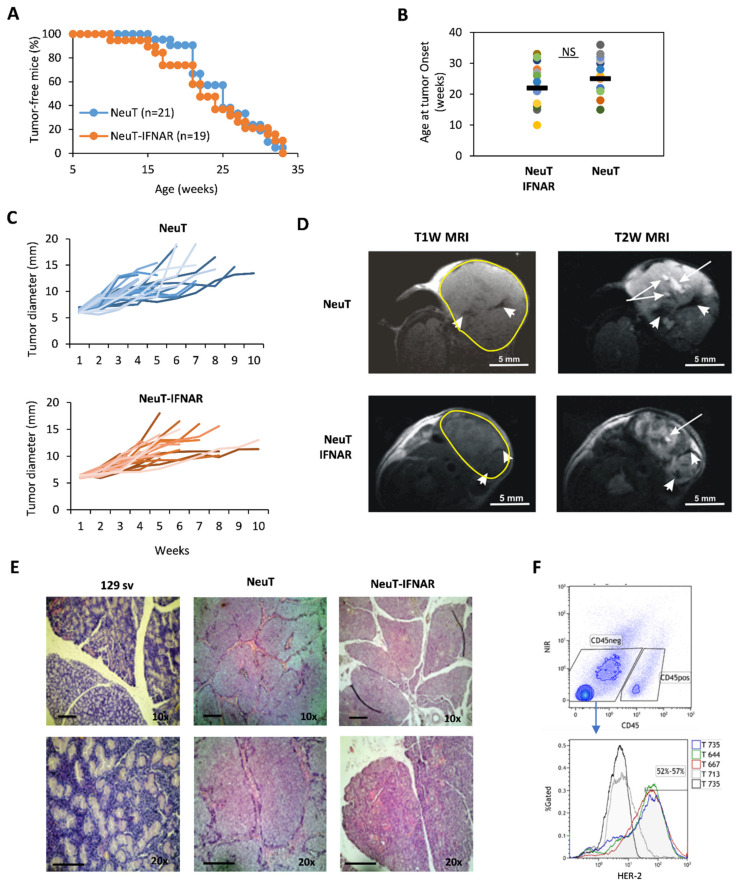
Characterization of NeuT and NeuT-IFNAR SDC models. (**A**) Tumor incidence in NeuT (blu line) vs. NeuT-IFNAR (orange line) mice. (**B**) Age of first tumor occurrence in both mouse strains. (**C**) Tumor development over time in NeuT (*n* = 25) and NeuT-IFNAR mice (*n* = 22). (**D**) Representative images of axial T1-weighted (T1W) and T2-weighted (T2W) MRI of NeuT and NeuT-IFNAR tumor mass of similar dimensions. White arrows in T2W images indicate necrotic areas; white head arrows in both T1W and T2W MRI indicate hemorrhagic regions. (**E**) Hematoxylin/Eosin staining of major salivary glands explanted from a naive 129sv and a 33 weeks old transgenic NeuT and NeuT-IFNAR mice. Scale bar: 100 µm (magnification 10× and 20×). (**F**) Surface staining with anti-c-ErbB2/c-Neu antibody after gating on viable CD45-negative cells in salivary gland tumor cell suspensions from NeuT and NeuT-IFNAR mice. Black-line histogram represents isotype control antibody-stained cells. Grey-line histogram represent staining of salivary gland from non-transgenic 129sv mice.

To this aim, NeuT and NeuT-IFNAR mice were monitored for salivary tumor development by weekly palpation, and subsequently assessed for tumor mass size by caliper measurement. Overall, tumor latency and incidence throughout the lifespan were similar in both mice strains (Figure 4A, log-rank test *p* > 0.05). The onset of the first palpable lesion occurred over a large time window (10–35 weeks of age), with a similar median age in both groups (Figure 4B). Tumor development occurred predominantly in the parotid glands and subsequently extended to the submandibular glands. No overt difference was observed between the size and growth kinetics of NeuT vs. NeuT-IFNAR-tumor masses (Figure 4C). Morphological characterization by in vivo T1-weighted (T1W) and T2-weighted (T2W) MRI revealed the presence of hyperintense areas in T2W images corresponding to necrosis and hypointense regions in T1W and T2W MRI attributable to hemorrhagic necrosis within tumors (as shown by white arrows and white head arrows, respectively, in Figure 4D) in both animal strains. Hematoxylin-eosin staining of major salivary glands explanted from 33 weeks-old NeuT-IFNAR-and NeuT mice showed advanced SDC with eosinophilic cytoplasm and a similar differentiation grade between the two mouse strains (Figure 4E). The Her-2 receptor was expressed on the surface of 52–57% of CD45-negative cells in the salivary gland tumors (Figure 4F) with no difference between NeuT and NeuT-IFNAR mice strains. Overall, the characterization of salivary tumors in NeuT vs. NeuT-IFNAR mice suggested that the lack of a functional IFN-I system did not significantly affect the development and progression of salivary tumors and confirmed that NeuT-IFNAR mice could be used as a specificity control in subsequent Sl-IFN-based therapeutic protocols.

### 3.4. Anticancer Effect of Combined CTX/Sl-IFN Treatment in SDC

To evaluate the synergistic effect of CTX and Sl-IFN in SDC, we preliminarily investigated the effect of a single CTX administration on tumor morphology and immune infiltrate composition. To this aim NeuT mice with advanced tumors (10 mm mean diameter approximately) were administered a single i.p. injection of CTX (100 mg/kg) and tumor masses were analyzed by MRI and flow cytometry. As shown in Supplementary Figure S1A, MRI analysis did not reveal alterations either in the anatomy or in average functional parameter values (ADC and perfusion component). Nevertheless, pixel-by-pixel analyses over entire lesions (whole-lesion analyses) showed sharper peak (increased kurtosis) in both ADC and perfusion component histogram 96 h after treatment (Supplementary Figure S1B,C), indicating more homogeneous tumors after treatment. The analysis of tumor infiltrate revealed the increased frequency of CD19^+^ lymphocytes and, among myeloid cells, of putative M-MDSC and DC into the tumor bed 96 h after CTX treatment (Supplementary Figure S1D,E), as previously reported [50].

**Figure 5:**
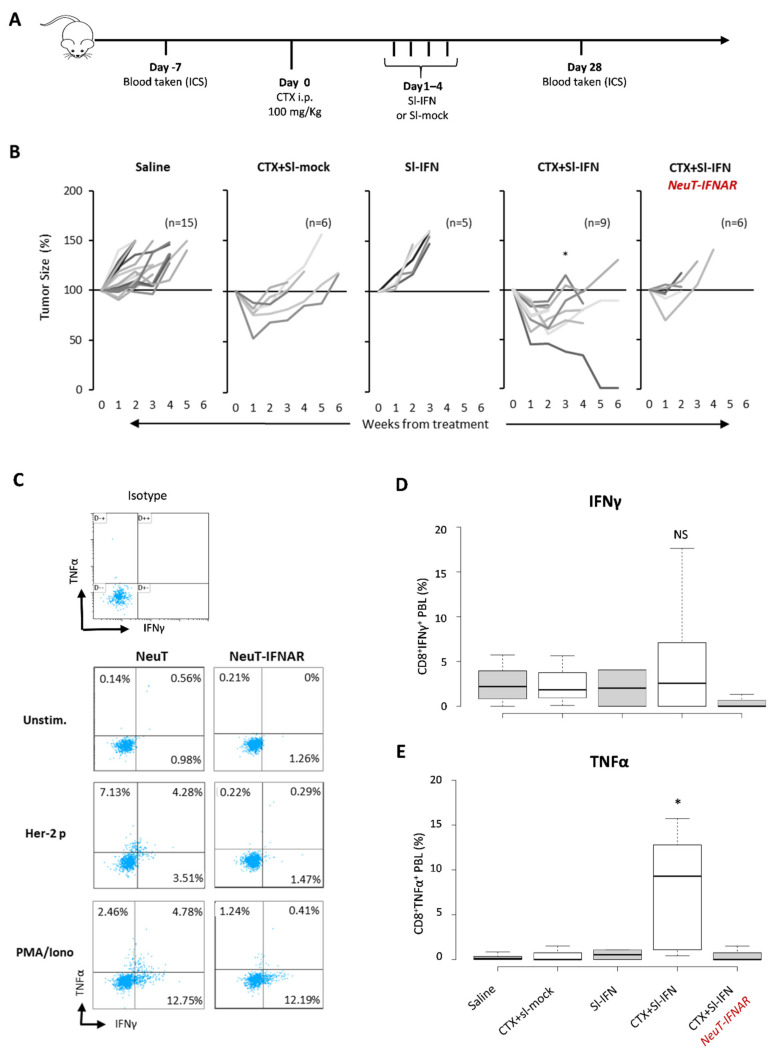
Therapeutic effect of combined CTX/Sl-IFN treatment in NeuT transgenic mice. (**A**) Schematic representation of the experimental design. (**B**) Percentage tumor size in NeuT 129sv mice treated with CTX and Sl-mock (*n* = 6) or with Sl-IFN (*n* = 5) or with a combination of CTX and Sl-IFN (*n* = 9) according to the schedule depicted above. One group of mice was treated with Saline as control (*n* = 15) and one group of NeuT-IFNAR was treated with CTX + Sl-IFN (*n* = 6) as specificity control. * *p* < 0.05 (only at week 3). (**C**) Representative dot plots showing the percentage of IFNγ^+^ and/or TNFα^+^ cells in CD3^+^ CD8^+^ T-gated cells from NeuT and NeuT-IFNAR mice treated with CTX and Sl-IFN. Blood samples were collected 28 days after treatment. Unstimulated, Her-2/Neu peptide-stimulated and PMA/Iono-stimulated PBL samples were stained and analyzed as described in Section 2. One representative dot plot from an isotype-stained control is also shown. (**D**) Box-Whisker plots depicting the percentage of IFNγ^+^CD8^+^ T lymphocytes and (**E**) the percentage of TNFα^+^CD8^+^T lymphocytes in the blood of mice treated as indicated after stimulation with Her-2/Neu peptide (435–443). Center lines show the medians; box limits indicate the 25th and 75th percentiles as determined by R software; whiskers extend 1.5 times the interquartile range from the 25th and 75th percentiles (*n* = 5).

To evaluate the therapeutic effect of CTX and Sl-IFN in SDC, NeuT mice were injected with CTX followed, one day apart, by four daily administrations of Sl-IFN (7.2 × 10^3^ U) or Sl-mock (Figure 5A). One group of mice received four doses of Sl-IFN, without CTX pretreatment, and another group was injected with saline as control. Treatment with a single injection of CTX + Sl-mock induced a transient reduction in tumor size (Figure 5B) similar to what was observed in other tumor models (Figure 3E and [50,51,54]).

Combination of Sl-IFN and CTX resulted in a remarkably reduced tumor size, which was far more efficient than single treatments (i.e., CTX + Sl-mock or Sl-IFN alone, Figure 5B). When the same combined treatment was applied to NeuT-IFNAR, the therapeutic effect of Sl-IFN was abrogated and the pattern of tumor growth in this mouse strain resembled the one in NeuT mice treated with CTX + Sl-mock (Figure 5B).

To evaluate the involvement of effector immune responses in the shrinkage of tumors following combined CTX/Sl-IFN therapy, we analyzed the frequency of polyfunctional TNFα/IFNγ-secreting CD8^+^T cells in the blood of NeuT and NeuT-IFNAR mice 28 days after treatment initiation. CD8^+^ T cells secreting either TNFα were detected in nearly all mice treated with CTX + Sl-IFN after stimulation with an immunodominant class-I-restricted Her-2 peptide (Her-2 *p*, Figure 5C), while TNFα/IFNγ double positive CD8^+^ T cells were observed only in a minority of mice. Minimal TNFα or IFNγ secretion in response to peptide stimulation was observed in mice from the other treatment groups (Figure 5D,E). At the same time point, no Her-2 specific IgG Abs were detected in the plasma of mice treated with CTX + Sl-IFN (data not shown), thus excluding an involvement of the humoral immune response in the observed therapeutic effect.

**Figure 6:**
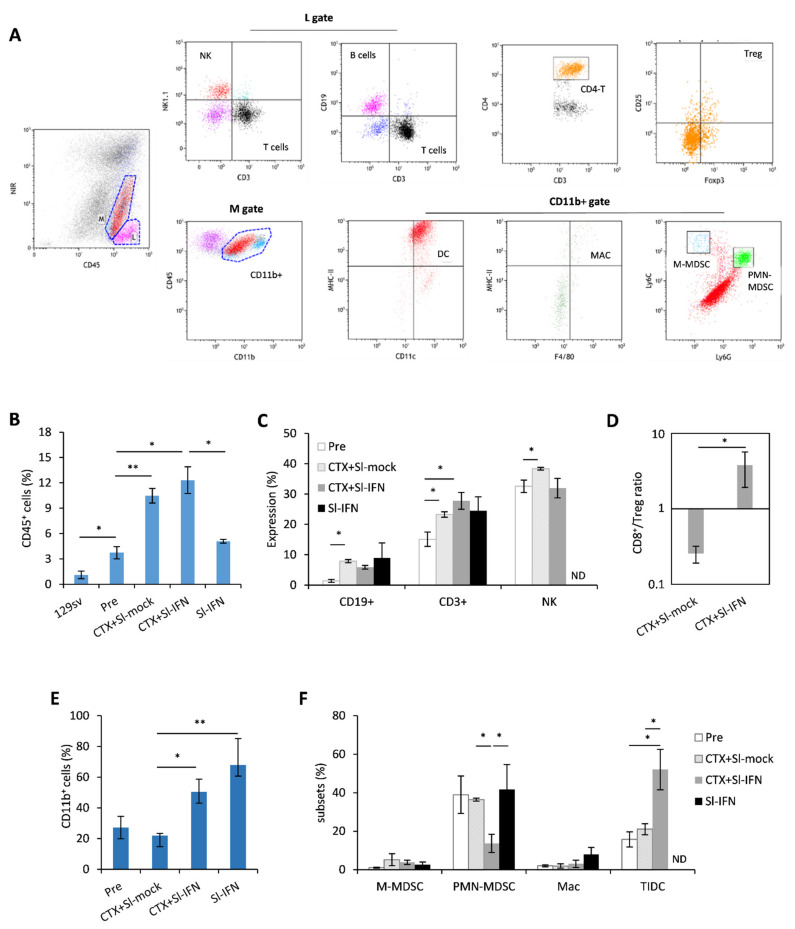
Analysis of leucocyte subsets in the tumor mass 22 days after treatment. (**A**) Representative dot plots of the gating strategy for lymphoid and myeloid subsets in tumor cell suspensions. (**B**) Percentage of CD45^+^ tumor-infiltrating cells in the tumor mass of NeuT 129sv mice treated with CTX and Sl-mock or Sl-IFN or with Sl-IFN alone as detailed in Section 2 (*n* = 4). (**C**) Percentage of the indicated lymphoid subsets in the tumor mass after gating on CD45^+^ SSC^lo^ cells. (**D**) CD8^+^ T/Treg ratio in the tumor. (**E**) Percentage of CD11b^+^ after gating on viable CD45^+^ cells, and of (**F**) tumor-infiltrating myeloid cell subsets. Myeloid subsets percentage is calculated after gating on CD11b^+^ cells. * *p* < 0.05; ** *p* < 0.01. ND, Not determined.

To corroborate the findings pointing to the involvement of the immune system in the antitumor response elicited by CTX + Sl-IFN, we phenotypically characterized the tumor infiltrate (Figure 6A–F). For this analysis, we selected a timepoint (Day 22) when the therapeutic effect of the single treatments was fading, while mice receiving CTX + Sl-IFN were experiencing further tumor shrinkage, implying possible immune-related effects. The neoplastic salivary glands of untreated NeuT transgenic mice displayed a higher percentage of CD45^+^ leucocytes when compared to the same tissue from non-transgenic 129sv. Treatment with CTX + Sl-mock or CTX + Sl-IFN induced an increase in CD45^+^ cells in the tumor tissue while treatment with Sl-IFN alone had no effect (Figure 6B). The analysis of tumor infiltrate composition showed that CD3^+^T lymphocytes, NK cells (Figure 6C) were the most abundant cell types in mice treated with CTX + Sl-mock and CTX + Sl-IFN among lymphoid subsets. Importantly, the combined CTX/Sl-IFN treatment induced an increase in CD8^+^T/Treg ratio in the tumor tissue as compared to CTX + Sl-mock (Figure 6D). Regarding myeloid subsets, which are maximally represented in mice treated with CTX + Sl-IFN and with Sl-IFN alone (Figure 6E), the therapeutic effect observed upon combined CTX + Sl-IFN seems to correlate with enhanced tissue resident DC (TIDC) and reduced polymorphonuclear-myeloid-derived suppressor-like cell (PMN-MDSC-like) tumor infiltration (Figure 6F). Changes in the frequency of M-MDSC-like and Mac among treatment groups were not observed (Figure 6F). In the spleen, CD3^+^ T lymphocytes were reduced in mice treated with either CTX + Sl-mock or Sl-IFN (Supplementary Figure S2) and CD8^+^T/Treg ratio was increased in mice treated with CTX + Sl-IFN with respect to mice treated with CTX + Sl-mock (Supplementary Figure S2). Among myeloid subsets, a decrease in putative M-MDSC and PMN-MDSC was observed in mice treated with CTX + Sl-IFN as compared to the other groups of treatment (Supplementary Figure S2).

Overall, these data suggest that combining Sl-IFN and CTX stimulates a local and systemic cellular immune response, whose principal actors are CD8^+^T cells and TIDC, leading to a prolonged reduction of tumor growth as compared to each single treatment alone.

## 4. Discussion

Sublingual treatments are characterized by a rapid onset of action and high patient compliance [37,38]. For this reason, they are primarily applied to diseases requiring immediate medication such as acute cardiovascular diseases and allergies [38]. However, since the sublingual route can also limit hepatic toxicity and proteolytic degradation associated with other administration routes [38], sublingual treatments hold promise for the treatment of chronic diseases such as cancer, which often requires high-dose or repeated drug administrations. Most importantly, sublingual delivery can stimulate both systemic and mucosal immunity [37], thus becoming particularly attractive for vaccination and other immunotherapies. While several studies have evaluated the sublingual route in allergies [40,55] and infectious diseases [56,57,58], few studies have been conducted in cancer settings [59,60,61]. Here, we show for the first time to our knowledge that Sl-IFN, as monotherapy or in combination with chemotherapy, stimulates antitumor immune responses in both transplantable tumor models and NeuT transgenic mice spontaneously developing SDC. Interestingly, the antitumor effect of Sl-IFN was comparable to the one induced by the same dose of IFNpti, although the lack of Mx1 upregulation in the PBL of mice treated with Sl-IFN suggests that sublingual and parenteral administration routes exploit different mechanisms. In fact, the induction of Mx1 expression following Sl-IFN occurred at the local more than at the systemic level, while IFNpti stimulated both the draining LN and the PBL. These findings confirm previous studies by Eid and colleagues showing that oromucosal IFN-I induces the expression of IFN-stimulated genes (ISG) in the lymphoid tissue of the oropharyngeal cavity, but not in PBMC, as opposed to parenteral injection [61]. In the same study, oromucosal IFN-I showed no significant effect on the number of circulating leucocytes nor in the number of granulocyte-macrophage colonies in the bone marrow up to ten days after IFN-I administration. On the contrary, we observed a statistically significant increase of CD3^+^CD8^+^, but not of CD3^+^CD4^+^, T lymphocytes in the blood of mice treated with Sl-IFN as compared to controls, 14 days after treatment. This finding is supported by studies from Sprent and colleagues showing that IFN-I (mainly α and β) exert indirect (via IL-15) bystander effects on CD8^+^CD44^hi^ memory cells in vivo, but not on CD4^+^CD44^hi^ [62]. Although we cannot confirm the “memory” phenotype of the lymphocyte subsets analyzed, the secretion of IFN-γ by leucocytes from IFN-treated animals following stimulation with MHC class I-restricted OVA peptide suggest that they are, at list in part, “bona fide” antigen-experienced CD8^+^T cells. In addition, it has been reported that cytokine-induced stimulation of CD44^hi^CD8^+^ cells in vivo applies not only to IFN-I (through IL-15), but also to IL-12, which operates through an intermediate production of IFN-γ released by NK cells [62], a cell subset abundantly present in sublingual tissue [63].

Several studies have shown that the administration of immunogens via the sublingual route can induce effector T cells in the cervical and smLNs, the primary draining LN for T cell priming after sublingual vaccination. T cell priming in the cervical LN is principally mediated by CD11c^+^ DCs [64]. Following sublingual vaccination, primed CD4^+^T lymphocytes enter the blood stream to migrate to other distant sites [37]. In our study, when given as single treatment (i.e., in the absence of any antigen), Sl-IFN produces a bystander magnification of already existing suboptimal immune responses, which mostly remain localized in the tissues adjacent to the administration site. Instead, when Sl-IFN is administered after chemotherapy, tumor antigens released in the surrounding tissue become available for presentation by tissue resident phagocytes [39,40], which, upon IFN-I encounter, become licensed for CD8^+^T cross-priming [65] and subsequently recirculate via the lymphatic vessels to the blood stream. In NeuT transgenic mice, this mechanism may be further enhanced by the anatomical localization of the tumor. In fact, CTX-induced tumor cell death occurring in the salivary glands enables leucocyte accrual from adjacent tissues, including IFN-activated APC from the sublingual mucosa, thus generating the optimal conditions for the stimulation of anticancer immune responses.

Hence, combined CTX/Sl-IFN treatment induced a long-lasting impairment of tumor growth in NeuT transgenic mice, but not in their IFNAR knock-out counterparts. This effect was accompanied by the emergence of circulating tumor-reactive effector CD8^+^T preferentially secreting TNFα rather than IFNγ upon antigenic stimulation. In a previous report, Brehm and colleagues showed that naive virus-specific CD8^+^ T cells preferentially produce TNFα after recognition of cognate ligand, while memory phenotype CD44^hi^ CD8^+^ T cells were able to produce both cytokines [66]. It is tempting to speculate that in NeuT transgenic mice treated with CTX and Sl-IFN, PBLs are enriched in naïve lymphocytes, which, however, are capable of immediate effector function, as a result of the transient lymphodepletion and subsequent homeostatic expansion of peripheral lymphocyte pools induced by CTX [54]. In addition to directly killing tumor cells and to the induction of homeostatic mechanisms, non-myeloablative CTX treatment can attenuate the tumor-suppressive environment by Tregs [51,67] and favor the recruitment of immune cell populations that are involved in tumor rejection [29,56,68,69]. Increased infiltration of CD8^+^ T cells in the tumor is associated with improved clinical responses in several malignancies, including Her2-expressing cancers [70], while the presence of PMN-MDSC in the tumor is associated with its pro-tumorigenic immunosuppressive phenotype [68]. In salivary gland tumors, however, there is limited and inconsistent information on the prognostic role of tumor immune infiltrates [71]. In adenoid cystic carcinoma, association of intratumoral CD8^+^ with CD1a^+^ cells associates with less recurrence and higher survival rates [72]. Although tumor infiltrates were hardly detectable in NeuT mice early after CTX administration, fine-tuned analyses such as histogram analyses of ADC perfusion component could detect subtle differences predictive of an immune response and more homogeneous tumors after treatment similarly to what already found after standard chemotherapy in ovarian cancer [69]. On the contrary, 22 days after treatment with CTX + Sl-mock and CTX + Sl-IFN the CD45^+^ fraction was highly enriched in tumor tissue. In particular, an increase in CD3^+^T and TIDC, and a reduction in putative immunosuppressive PMN-MDSC and Tregs, as testified by the increased CD8^+^T/Treg ratio, was observed in the spleen and tumor tissues of NeuT mice treated with CTX + Sl-IFN as compared to saline-treated controls. These results suggest that in mice treated with CTX + Sl-IFN the balance between immunogenic and immunosuppressive cell subsets is in favor of the former.

The combination of chemotherapy and immunotherapy, including IFN-I, has already proven effective in inducing effective anticancer responses in a number of cancer settings [27,28,29,30,31,32,73,74]. In a previous study from our group, the combination of non-myeloablative doses of CTX with the adoptive transfer of tumor-immune cells and immunoglobulins induced the complete regression of large established breast tumors spontaneously developed in NeuT transgenic mice [26]. More recently, active immunization with NeuT-expressing viral vectors as a single agent or in combination, has also been explored in transplantable salivary gland tumor models with encouraging results [23,24]. Notably, the therapeutic outcome of the combined CTX and Sl-IFN in the NeuT transgenic SDC model is promising in view of novel therapeutic settings involving the routing of immunotherapy to target specific anatomical districts. In fact, the expression of an activated oncogene provides a significant advantage for the development and progression of spontaneous neoplastic lesions. In addition, NeuT transgenic mice are immune tolerant against the self-oncogene, which makes it extremely difficult to elicit an effective antitumor immune response in this model. Although the immune status of NeuT transgenic male mice has not been extensively characterized in the present study, our data show that untreated mice lack detectable spontaneous cell-mediated immune response against Her2. This finding is in line with the tolerogenic phenotype reported in similar Her2-expressing mouse models [75,76,77]. Of note, the tumor regression observed in mice treated with CTX and Sl-IFN, only transiently induced by CTX and Sl-mock, persisted for several weeks, thus suggesting the elicitation of an effective and long-lasting antitumor immune response overcoming the pro-carcinogenic signal of Her2 in tumor cells and immune tolerance. In this light, the increase in Her2-specific TNFα-secreting CD8^+^T cells observed in CTX + Sl-IFN-treated mice testifies to the efficacy of the combined treatment in breaking tolerance against a self-antigen. The lack of detectable Her-2 IgG Abs in NeuT mice treated with CTX + Sl-IFN suggests that cell-based immunity is preferentially elicited by this treatment combination. The advanced tumor stage and the lack of a co-administered exogenous antigen may have contributed to the low humoral response observed in our study. On the other hand, we may speculate that in this therapeutic setting, CTX-induced intratumoral CD19^+^ cells play important roles in immune responses that extend well beyond their canonical functions as antibody producers and include cytokine production, antigen presentation, costimulation, and contribution to lymphoid tissue development, thus concurring to optimal antitumor immunity [78].

Overall, these results support the notion that immune-mediated regression of spontaneous Her2^+^ SDC tumors can be achieved through multiple immunomodulatory activities induced in vivo by the combined treatment of CTX and Sl-IFN. We believe that Sl-IFN holds promise for the treatment of established tumors, although further studies are needed to fully explore the biological mechanisms and the potential efficacy of combination therapies based on Sl-IFN in different animal models and in human settings.

## Data Availability

Not Applicable.

## Supplementary Materials

The following are available online at https://www.mdpi.com/article/10.3390/cells10040845/s1, Figure S1, Figure S2: Analysis of leucocyte subsets in the spleen 22 days after treatment.
