# Supplementary figures and images for: Anticancer Effects of Sublingual Type I IFN in Combination with Chemotherapy in Implantable and Spontaneous Tumor Models
Source: Cells. 2021 Apr 8;10(4):845. doi: 10.3390/cells10040845 (PMC8068355; doi:10.3390/cells10040845)

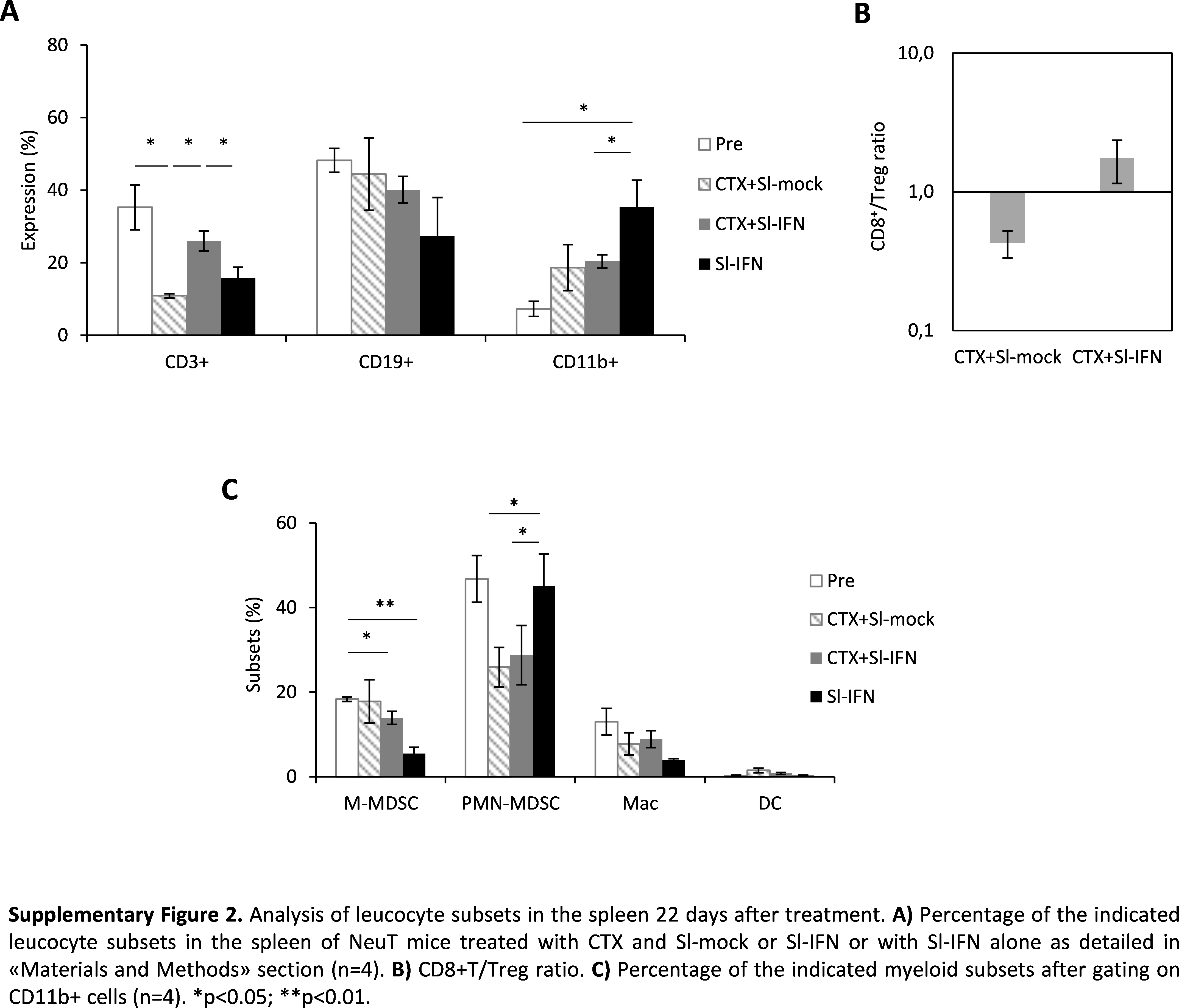

Supplement: Supplementary file 1 [file cells-10-00845-s001.zip › supplementary figure 2.tif]

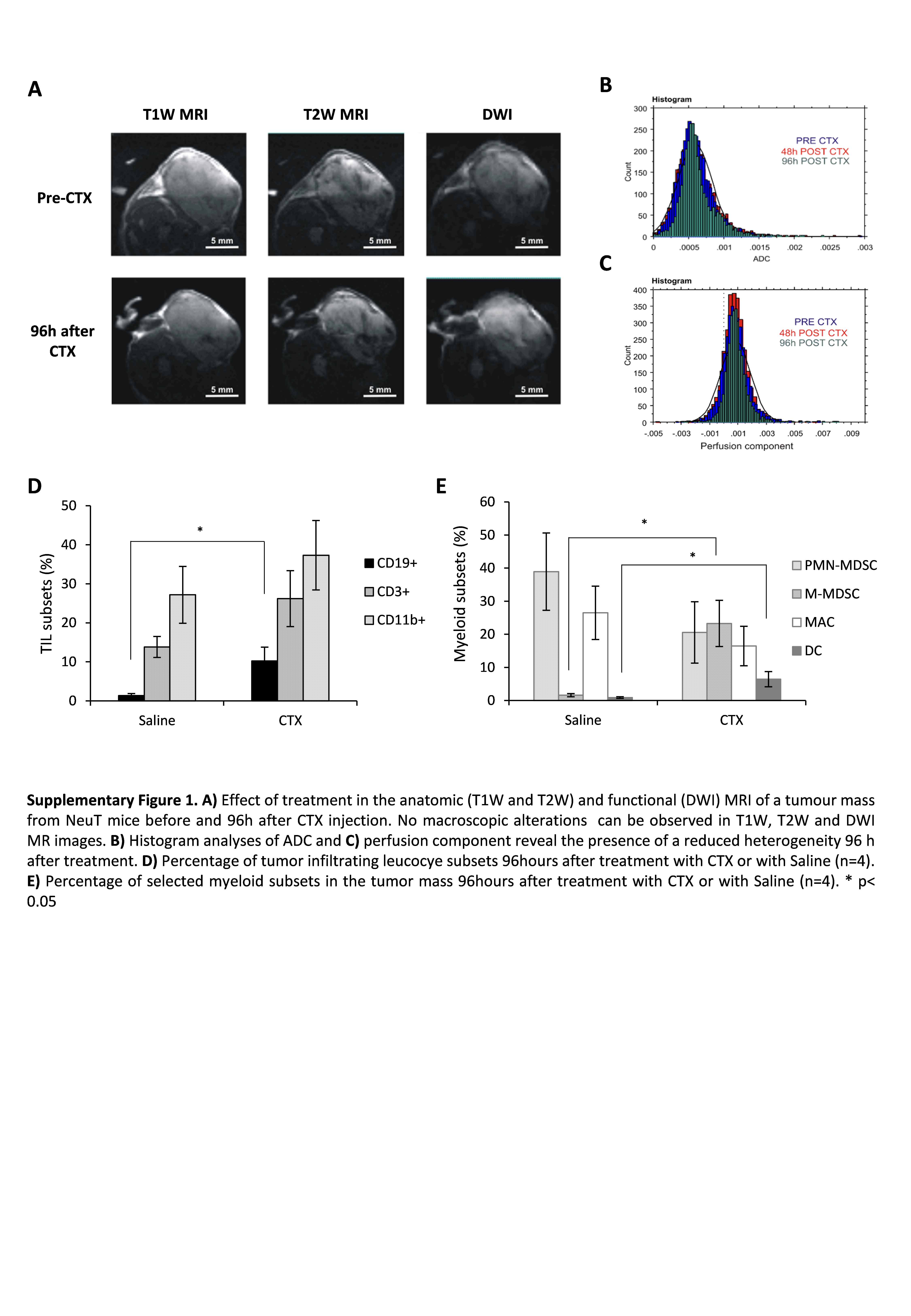

Supplement: Supplementary file 1 [file cells-10-00845-s001.zip › supplementary figure 1.tif]
